# Prognostic evaluation of colorectal cancer using three new comprehensive indexes related to infection, anemia and coagulation derived from peripheral blood

**DOI:** 10.7150/jca.42409

**Published:** 2020-04-06

**Authors:** Yalun Li, Huizhe Wu, Chengzhong Xing, Xiaoyun Hu, Fangxiao Zhang, Yangjie Peng, Zeyu Li, Tingting Lu

**Affiliations:** 1 Department of Anorectal Surgery, First Affiliated Hospital of China Medical University , Shenyang, Liaoning, China; 2 Department of Pharmacology, School of Pharmacy, China Medical University, Shenyang, Liaoning, China; 3 Department of Intensive Care Unit, First Affiliated Hospital of China Medical University, Shenyang, Liaoning, China

## Abstract

**Background**: Many indicators of peripheral blood in routine blood test (BRT) results of colorectal cancer (CRC) patients are related to prognosis. Currently, indexes such as NLR (Neutrophil-to- Lymphocyte Ratio), PLR (Platelet-to-Lymphocyte Ratio) and LMR (Lymphocyte-to-Monocyte ratio) evaluate the survival risk of patients by assessing the inflammatory - immune status of CRCs. These indexes are more comprehensive and accurate than independent estimates. We hope to design more effective indexes through fully considering the correlation and significance between BRT indicators and prognosis, so as to play a guiding role in clinical malignant estimation of CRCs.

**Methods**: 701 CRCs in training set and 256 CRCs in test set were included in the study samples, and their clinical data, tumor pathology results and peripheral blood routine results were collected. The prognosis, progression, and survival status of all patients were determined after follow-up. Above data were used for statistical analysis and designing new indexes.

**Results**: It was found that high NE, MONO, RDW-CV/SD and PLT in peripheral blood indicated poor prognosis of DFS and OS. Conversely, CRCs with postoperative tumor progression or death had lower LY, EO, RBC, HGB, HCT, MCV, MCH, MCHC, PDW, and P-LCR. IRR, ARR and CRR related to infection, anemia and coagulation were designed respectively using the largest AUC indicators (P<0.05) selected by ROC curve. The formula: IRR= (NE*MONO)/(LY*EO); ARR= (HGB*MCHC)/RDW-CV; CRR=PLT/PDW. Results of Kaplan‑Meier survival analysis and multivariate COX proportional hazard analysis adjusted for age, gender, TNM stage, infiltration, adhesion showed IRR, ARR, CRR were all able to be used as the evaluation standard of survival of CRC. The result was also authenticated in the test set.

**Conclusion**: We designed three different prognostic indexes of colorectal cancer, IRR, ARR and CRR, which could be used as risk indicators of CRC prognosis, tumor progression and survival.

## Introduction

Colorectal cancer (CRC) is one of the most common cancers worldwide and is associated with a high mortality rate due to its rapid progression^1^. Generally, postoperative pathological analysis results are used to evaluate the degree of malignancy in terms of TNM-stage-based invasion depth, lymph node metastasis, pathological type and tumor growth pattern, so as to predict the prognosis and formulate auxiliary treatment programs^2^, ^3^. With the deepening of biomarker research, various types of indicators had been found to be related to the biological characteristics even prognosis survival of colorectal cancer, including genetic molecules, blood parameters and even nutritional indexes^4^-^6^. Therefore, the exploration of reliable and concise indexes may help doctors more accurately to evaluate CRC malignancy and screen for prognosis, which is of great significance for clinical treatment.

Peripheral blood test is commonly used in clinical practice. Blood routine test (BRT), as one of the most basic peripheral blood biochemical tests, can quickly and accurately detect the values of blood components such as white blood cells (WBC), lymphocytes (LY), red blood cells (RBC), hemoglobin (HGB) and platelets (PLT), as well as other related indicators, in order to effectively indicate abnormalities of infection, anemia and cruor^7^-^9^. In recent years, a wide variety of blood indicators with different changes were concerned and discussed in the study of malignant tumor diseases including CRC: Qian W, et al. reported that the post-/pre-treatment MPV (Mean Platelet Volume) ratio were prognostic factors for OS in resectable CRCs^10^; Preoperative neutrophil-lymphocyte ratio (NLR) and red blood cell distribution width (RDW), especially the independent prediction of NLR, were confirmed as effective biomarkers for clinical diagnosis and prognosis evaluation of esophageal cancers^11^; In patients with pathologic stage I non-small cell lung cancer undergoing surgical resection, high LY and PLT count from peripheral blood could provide poor prognostic value independently^12^; Even the ratios between indicators were developed into new indexes, which had fairly good prognostic significance, including NLR, Platelet-to-Lymphocyte Ratio (PLR), Lymphocyte-to-Monocyte ratio (LMR).^13^-^16^.

Compared with the traditional pathological test of tumor lesions, peripheral blood biochemical test has the great advantages of quick and simple sample acquisition, low collection cost, minimal trauma and preoperative detection, which should be paid more attention to in research. We reported that RDW-CV (Red Cell Distribution Width-CV) combined with CEA can effectively predict the poor prognosis of CRC^17^. Similarly, it was observed that LY, HGB, MCV (Mean Corpuscular Volume), MCH (Mean Corpuscular Hemoglobin), MCHC (Mean Corpuscular Hemoglobin Concentration), PDW (Platelet Distribution Width) and some other indicators in peripheral blood of CRCs often presented comprehensive abnormalities, which aroused our strong interest. Therefore, we boldly speculate that innovative indexes that have a specific relationship with prognosis of colorectal cancer can be obtained by calculating blood routine indicators of patients with CRC. We conducted the following retrospective study in the hope of verifying new prognostic indexes and comparing them with NLR, PLR and LMR popular comparative indexes, so as to provide clinical guidance for the assessment of CRC prognostic risk.

## Materials and Methods

### Ethics statement

The First Hospital of China Medical University and the Medical Ethics Committee of China Medical University approved this study. Due to the retrospective nature of the study, the First Hospital of China Medical University and the Medical Ethics Committee of China Medical University waived the need of written informed consent by the patients. All the samples were anonymous.

### Patients and methods

#### Study Population

Patients with CRC who received systemic therapy including laparoscopic surgeries with adjuvant postoperative chemotherapy were recruited between January 2012 and December 2015 at the department of Anorectal Surgery, First Affiliated Hospital of China Medical University. All patients accepted preoperative blood biochemical tests (BRT, blood electrolyte, hepatorenal function, blood gas analysis), colonoscopy, contrast-enhanced CT examination of lung and abdomen, and other necessary cardiopulmonary function assessment. Serum tumor marker (CEA, AFP, CA12-5, CA15-3, CA19-9) in peripheral blood, regular CT scans and colonoscopy during follow-up were used to detect tumor metastasis and/or progression. The study population was selected according to the following criteria and followed up to September 2018.

The inclusion criteria: I) Pathological diagnosed with CRC; II) Complete blood samples were approved for experimental analysis; III) Patients insisted on rechecking rigorous post-operative review and adjuvant chemotherapy in accordance with the National Comprehensive Cancer Network (NCCN) guidelines (www.nccn.org)^18^; IV) Follow-up compliance was good and detailed clinicopathological data were available.

The exclusion criteria: I) Incomplete patient information and/or loss of follow-up; II) No complete preoperative result of BRT; III) Preoperative adjuvant chemotherapy and/or radiotherapy; IV) Postoperative review and adjuvant treatment were not appropriately accepted; V) Patients with history of other serious diseases that affect survival outcomes possibly, such as myocardial infarction, cerebral infarction, high risk of hypertension and/or infectious diseases, etc.; VI) Severe postoperative complications including intestinal fistula, anastomotic obstruction, and pulmonary infarction.

**Figure 1:**
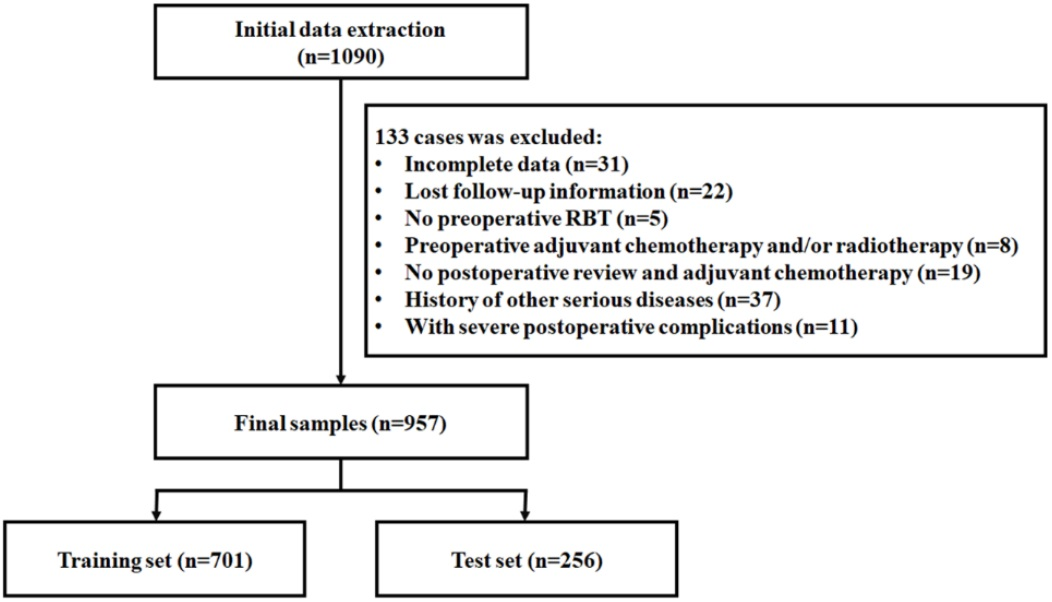
Screening process of sample patients.

Finally, 957 CRC patients were collected. All laparoscopic surgeries were presided over by two experienced surgeons and operation, to ensure there were no obvious differences between surgeries. The total samples were divided into training set (701 samples) and test set (256 samples) according to different surgeons.** (Figure 1)**

#### Blood Biochemistry

All peripheral blood samples obtained from patients with fasting state were examined by laboratory department of the First Affiliated Hospital of China Medical University through blood cell analyzer (Sysmex XE-5000, Japan) one week before the operation.

#### Tissue Pathology

Postoperative complete resection of tumor specimens was sent to the second tumor institute of the First Affiliated Hospital of China Medical University for Pathological Examination in 12 hours. The TNM stage involved infiltration depth and lymph node metastasis, pathological type, differentiation degree, tumor growth pattern and morphology were reported by qualified professional pathologists according to the latest AJCC cancer manual^19^. Intraoperative laparoscopy was used to observe the adhesion between tumor and surrounding organs.

#### Methods

Complete clinical and pathological characteristics of the sample patients were collected for collation and statistical analysis, including gender, age, BRT results and pathological description. All patients were required to follow-up and review strictly after treatment for tumor recurrence and survival. Disease free survival (DFS) was based on the last CT and colonoscopy to assess the progress of CRC; Overall survival (OS) was based on whether the patients were live or not.

### Statistical analysis

Statistical analysis was conducted by SPSS 24.0 (Chicago, IL, USA) and Graphpad Prism 7.0 (Graphpad Software, CA, USA). The classification data were analyzed using the Pearson chi-square test, continuous variables were tested by spearman two-variable correlation test and the linearity was analyzed by the logistic regression analysis. ROC curve was used to evaluate the sensitivity, specificity and the area of AUC. The survival rate was calculated using Kaplan‑Meier method test and the multivariate COX proportional hazard regression model was used to evaluate the association of multiple variables. A *P* value of less than 0.05 (*P* <0.05) was considered statistically significant.

## Results

In the training set, there were a total of 701 patients with an average age of 61.8 years included 342 young and 359 old people. Male/female patients and colon/rectal cancer were 428/273 and 227/474, respectively. The right and left colon cancers (89/138) occurred in the cecum/ascending colon/hepatic convolutions and splenic convolutions/descending colon/sigmoid colon. In terms of tumor TNM stage, there were 111, 292, 262, 36 cases in stage I to IV. In detail, 450 cases of tumor invaded serous membrane (T4), among which 103 cases were found tumor adhered to peripheral tissue or organs during surgery (T4b); In addition, 279 cases of lymphatic node metastasis (N1, N2) and 36 cases of lung and/or liver metastasis were found (M1). Described in pathological reports, 130/384/187 cases of poor/medium/well differentiation, 552 cases of adenocarcinoma and 149 cases of non-adenocarcinoma pathological types such as mucinous adenocarcinoma and signeta-ring cell carcinoma (SRCC) with higher malignancy were distinguished. Moreover, 554 and 411 CRCs showed ulcerative or invasive form of tumor morphology and growth pattern. By the end of follow-up, 184 cases of cancer progression were found, and 178 people died. Average DFS and OS time were 989.3 and 1199.8 days. BRT results were shown in the supplementary materials. **(Table 1, Table S1)**

### Indicators of peripheral blood were correlated with multiple CRC characteristics

Bivariate correlations between continuous variables of BRT outcomes and clinical, pathological characteristics of CRCs were analyzed, and many statistically significant results were obtained. According to the content reflected by peripheral blood indicators, they were divided into three parts: inflammatory, anemia and coagulation. In addition to differentiation, various indicators of BRT were closely related to the clinical, pathological and survival of CRC. In the peripheral blood of elderly CRCs, the values of LY, BASO (Basophilic Granulocyte), RBC, HGB, HCT, MCH and MCHC were lower, while the values of RDW-CV /SD were higher; The values of WBC, NE, MONO, EO, RBC, HGB, HCT, MCV, MCH and MCHC were higher in male patients, while those of RDW-CV, PLT, P-LCR (Platelet-Larger Cell Ratio), PCT (Platelet Cell Thrombocytocrit) and MPV were higher in female patients; Compared with colon cancers, the peripheral blood of patients with rectal cancers showed higher LY, RBC, HGB, HCT MCV, MCH, MCHC and lower RDW-CV, PLT, PCT; The left colon cancer patients had lower LY; CRCs with poorer TNM stage showed high NE, MONO, RDW-CV, PLT and low LY, HGB, MCV, MCH, MCHC, PDW; Specifically, NE, MONO, BASO, PLT, PCT were higher and LY, HGB, HCT, MCV, MCH, MCHC were lower in CRCs with T4, while CRCs with N1/2 were only associated with lower LY, and WBC, NE, MONO were higher and RBC, HGB, PDW were lower in M1 patients; Peripheral blood of non-adenocarcinoma CRC patients tended to have the characteristics of low levels of HGB, HCT, MCV, MCH and MCHC; CRC patients with ulcerative/invasive morphology tumors showed higher PLT, while patients with protuberant type tumors showed higher PDW, P-LCR and MPV in peripheral blood; Tumors of ulcerative/invasive growth pattern were associated with lower EO; Patients with intraoperatively found adhesion between tumors and surrounding tissues and organs showed high values of WBC, NE, MONO, RDW-CV, PLT, PCT and low values of LY, RBC, HGB, HCT, MCV, MCH, MCHC, PDW, P-LCR, MPV; In the prognostic survival correlation results as the main research objective, we found that higher NE, MONO, RDW-CV, RDW-SD (Red Cell Distribution Width-SD), PLT could lead to worse prognosis of DFS and OS; on the contrary, high blood values of LY, EO, RBC, HGB, HCT, MCV, MCH, MCHC, PDW could indicate good DFS and OS survival, while P-LCR was only correlated with good DFS, but not with OS. **(Table 2, Table S2)**

### Screening effective new peripheral blood indexes for CRC prognostic evaluation

**Figure 2:**
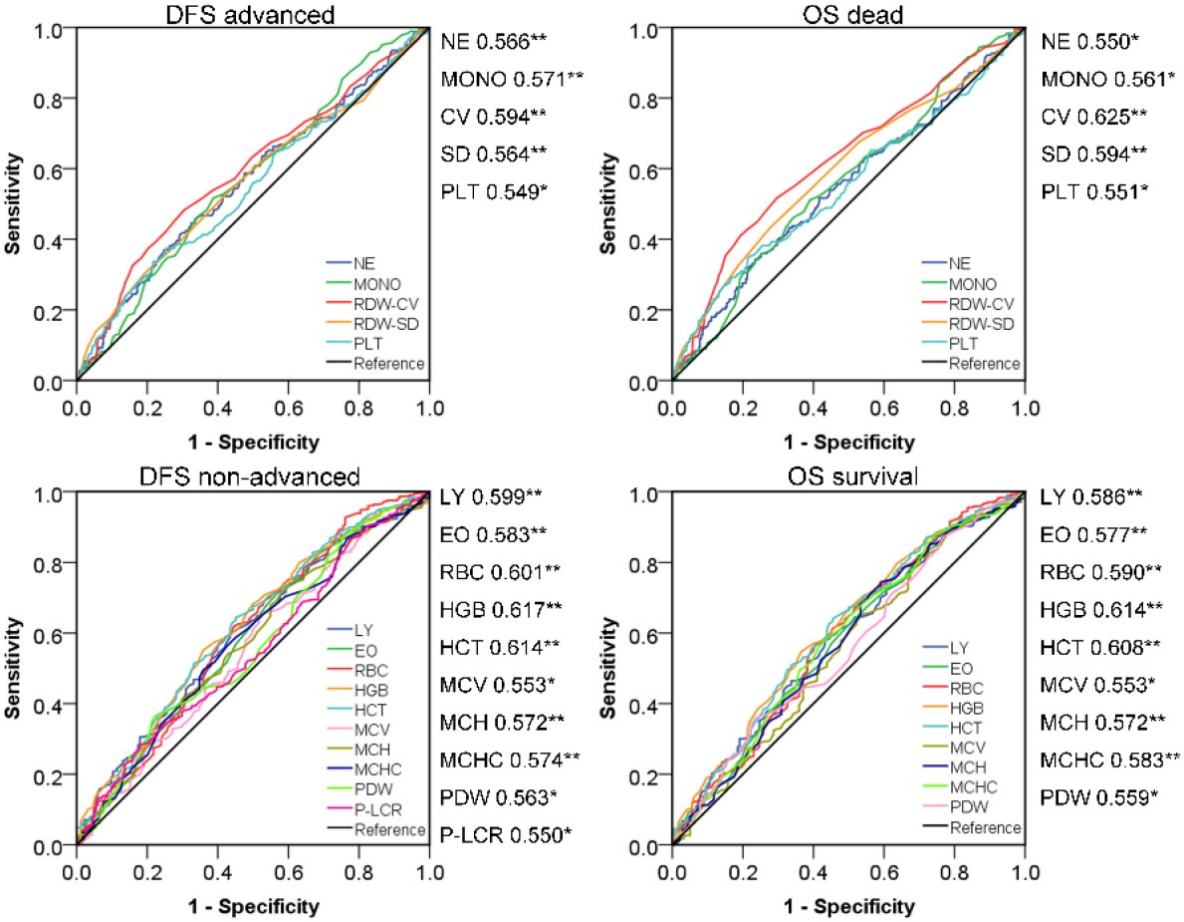
Prognosis survival ROC curve of peripheral blood indicators.

**Figure 3:**
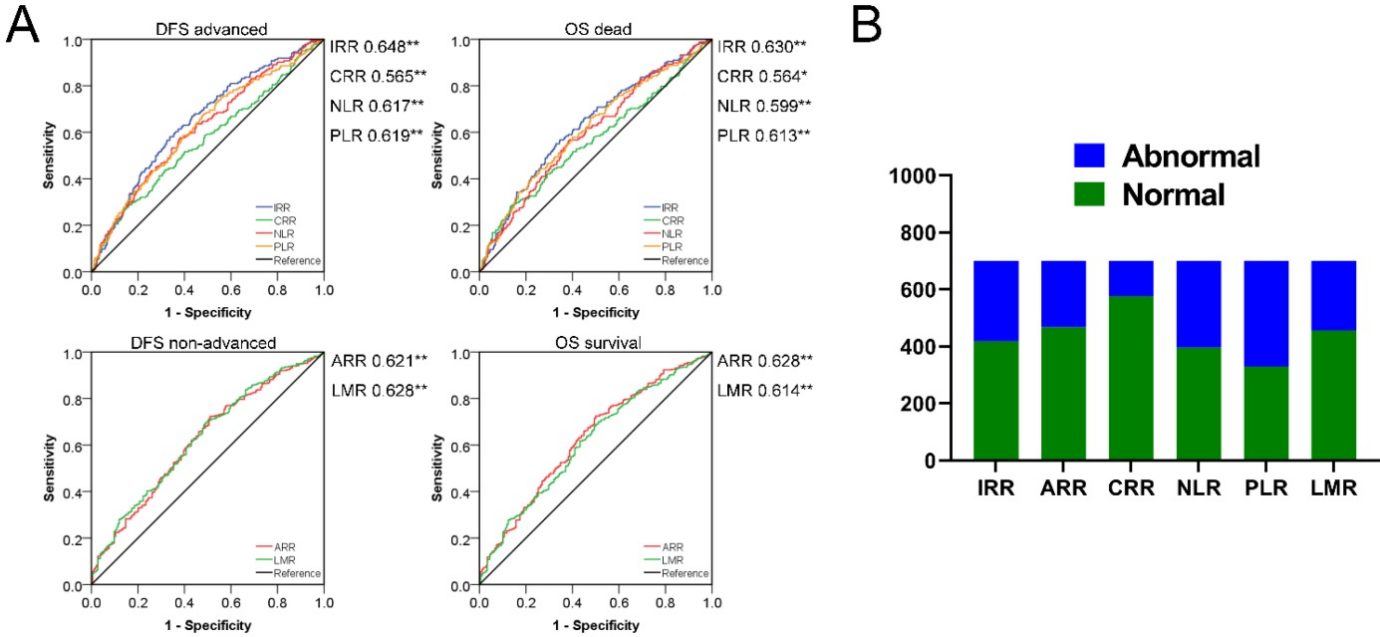
Divide CRCs into the abnormal and normal group using the cutoff value of IRR, ARR, CRR, NLR, PLR and LMR: **A.** Prognosis survival ROC curve of indexes. **B.** Distribution of different biochemical indexes.

According to bivariate correlation results, we found that the indicators in peripheral blood were very closely related to the following clinical and pathological characteristics: Age, gender, TNM stage, infiltration, adhesion, DFS and OS. Age and gender were used as covariates for subsequent studies due to differences in the health base range. ROC curves with statistical correlation between indicators of BRT and DFS/OS were drawn. On the premise of *P*<0.05, the AUC area and the content reflected were referred to to select the appropriate indicators comprehensively.** (Figure 2)** The contents reflected by RBC/HGB/HCT showed no difference and tended to be the same, as in MCH/MCV/MCHC and RDW-CV/SD. Therefore, we extracted the most prominent indicators in the AUC region of positive and negative correlation in three different connotations respectively, and constructed the following three innovation indexes: IRR (inflammatory related ratio), ARR (anemia related ratio), CRR (coagulation related ratio). Specific formula was as follow: IRR= (NE*MONO)/(LY*EO); ARR= (HGB*MCHC)/RDW-CV; CRR=PLT/PDW. Finally, we obtained IRR, ARR and CRR as potential indexes with cutoff values of 8.91, 3204.13 and 27.22 respectively in ROC curve draw. It is noteworthy that the association between ARR and poor prognosis is reversed, thus 282,233,125 cases with abnormal scores and 419,468,576 cases with normal scores were confirmed. In a similar way, we also calculated the cutoff value of NLR, PLR, LMR (2.24, 129.25, 3.66) to obtained the 305, 371, 244 abnormal cases and 396,330,457 normal cases. Surprisingly, IRR was better than all other indexes in terms of prognosis of DFS and OS; ARR was more effective than NLR and PLR in DFS prognosis assessment, and also exceeded the three comparative indexes in OS correlation; Although CRR AUC area was greater than 0.5, it did not show strong indications for poor prognosis. **(Figure 3)**

### IRR/ARR/CRR can effectively evaluate the risk of CRC prognosis

**Figure 4:**
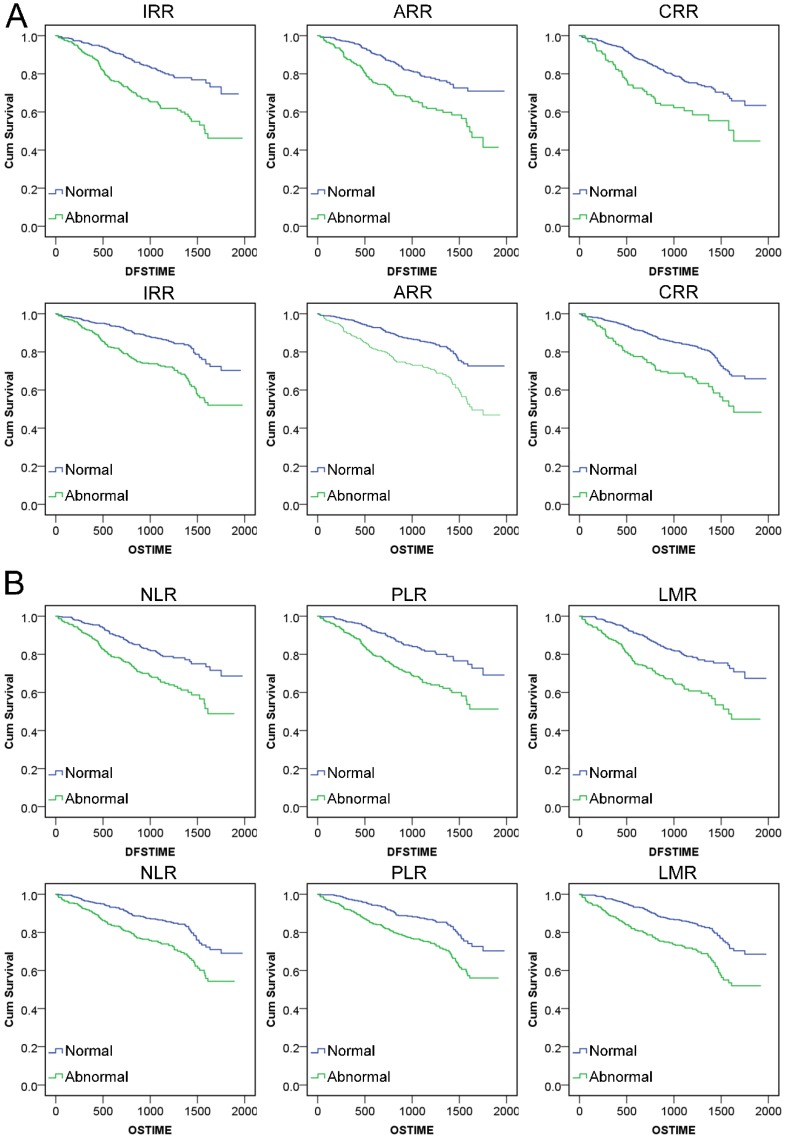
Kaplan‑Meier survival analysis curve of indexes: **A.** DFS and OS survival for IRR, ARR and CRR. **B.** DFS and OS survival for NLR, PLR and LMR.

Through calculating Kaplan‑Meier survival analysis, we found that abnormal conditions of IRR, ARR, CRR and NLR, PLR, LMR all could reflect shorter survival time (P<0.001). The mean DFS and OS time of CRCs with these indexes in the normal range was much longer than that in patients with abnormal level. **(Figure 4, Table S3)** We analyzed the logistic regression analysis between new indexes and TNM stage, infiltration, adhesion, DFS, OS for the CRCs with different level scores of six indexes. According to the results, all six indexes were related to TNM stage, infiltration, adhesion, DFS and OS, which clarified the adjustment variables for COX analysis. **(Table 3)** The multivariate COX proportional hazard analysis was used to evaluate the effectiveness of indexes adjusted for age, gender, TNM stage, infiltration and adhesion. The results showed that all six indexes were statistically significant in relation to the prognosis of DFS and OS. **(Table 4)**

### Verification of the validity of the new indexes in the test set

**Figure 5:**
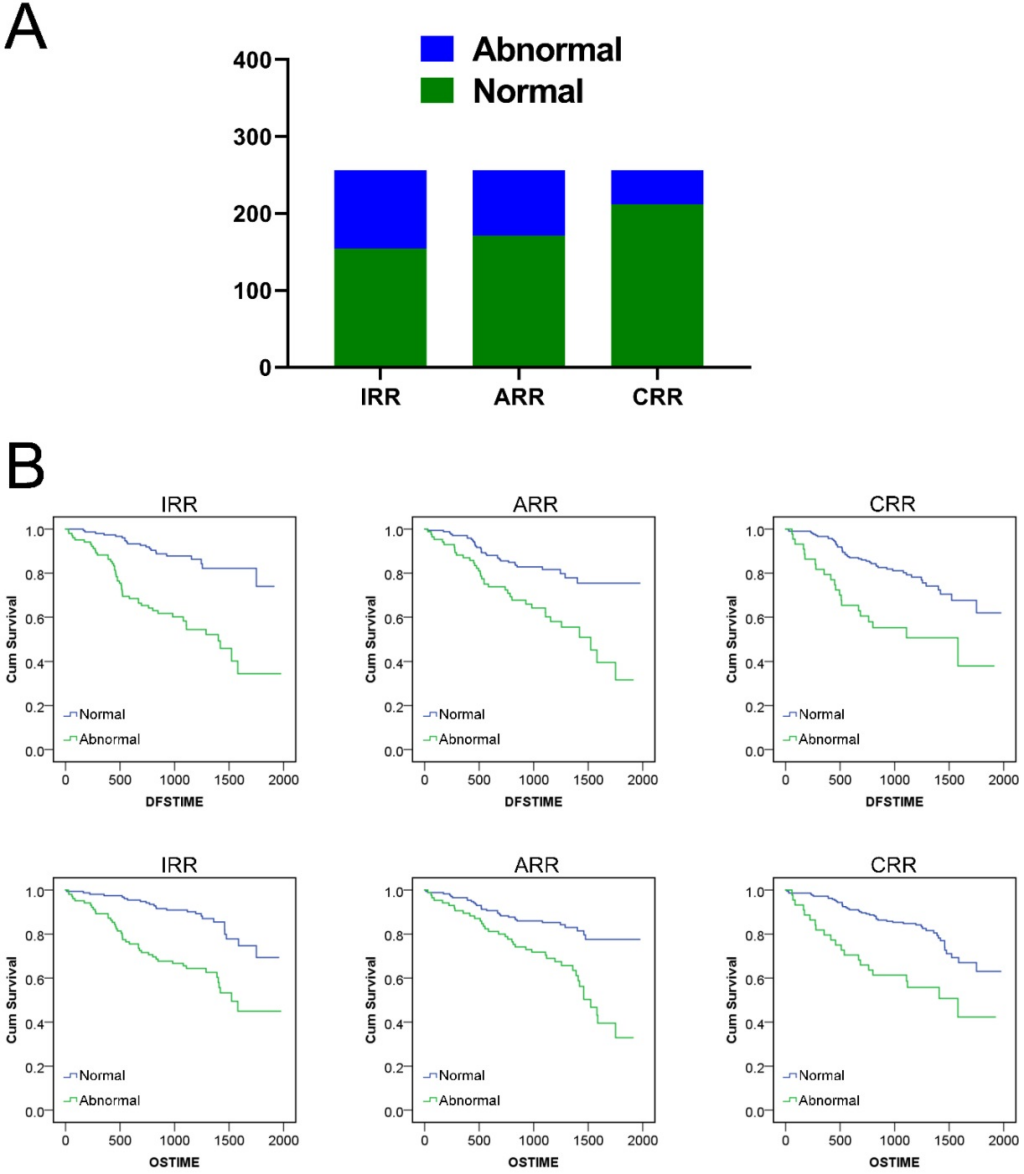
Survival analysis of IRR, ARR and CRR in test set: A. Distribution of three indexes in test set. B. DFS and OS Kaplan‑Meier survival analysis for three indexes.

Finally, we validated the prognostic risk factor role of IRR, ARR and CRR with the test set of 256 CRCs. According to the statistics, a total of 154, 171, 212 and 102, 85 and 44 CRCs were included in normal and abnormal groups according to the cut-off critical value obtained in the previous study. Kaplan‑Meier survival analysis results showed that normal CRCs with IRR, ARR and CRR had a longer postoperative DFS and OS time than the abnormal group. **(Figure 5, Table S4)** The multivariate COX proportional hazard analyses adjusted for age, gender, TNM stage, infiltration and adhesion proven that all three indexes were valid prognostic risk predictors for CRCs, which were consistent with those of the training set. **(Table S5)**

## Discussion

At present, the main treatment for CRC has developed into a comprehensive treatment based on surgical intervention. Patients with CRC must receive BRT as one of the basic peripheral blood tests before surgery. Doctors assess the patient's health and surgical risk based on blood readings of infection, anemia and coagulation. The new indexes derived from the indicators of peripheral blood can comprehensively consider multiple factors and more completely evaluate the abnormalities of a certain aspect of human body, among which the most widely discussed indexes for CRC are NLR, PLR and LMR^20^-^22^. In this retrospective study, multiple peripheral blood indicators could independently indicate the risk of CRC prognosis. On this basis, we further classified the indicators according to infection-related, anemia-related and coagulation-related, selected appropriate positive and negative correlation factors for ratio. Finally, innovative IRR, ARR and CRR indexes were successfully designed and verified to indicate CRC prognostic risk effectively. The ROC curve calculated the AUC area to evaluate the sensitivity and specificity of the indexes, and the results were surprisingly suggested that IRR was the most sensitive and specific one in risk assessment of DFS and OS; Abnormal ARR also performed better in relation to poor prognosis of OS than NLR, PLR and LMR, while the role of CRR was not satisfactory. KM and COX analysis after the cutoff value was used to distinguish CRC patients into normal group and abnormal group suggested that: abnormalities of three indexes all could indicate poor prognostic outcomes. Since we selected positive/ negative indicators with the largest AUC area for the ratio optimal prognostic sensitivity and specificity and drew up new indexes from three different perspectives, the role of IRR, ARR and CRR in poor prognosis screening was more convincing and explanatory. Therefore, we suggest that these three innovative indexes had potential to be new CRC prognostic criteria.

CRC patients are often accompanied by a low level of systemic inflammatory response, including an increased proinflammatory cytokines and acute phase proteins entering the circulation, which ultimately leads to systemic malignant tumor wasting cachexia in various aspects^23^. Numerous articles had analyzed the relationship between inflammatory cytokines and the CRC progression and prognosis. Although the specific mechanism of action is still controversial, but it becomes a consensus among many researchers that the inflammatory response that accompanies cancers can induce and promote tumor progression^24^-^26^. It is now widely accepted that: Many tumor-promoting effects of "smouldering" inflammation in the tumor microenvironment play the carcinogenic role through external pathways (inflammatory environment that increases the risk of cancer) or internal pathways (genetic changes), which ultimately affect the proliferation and survival of malignant cells, promote angiogenesis and metastasis, destroy adaptive immune response, and change the response to hormones and chemotherapy drugs^27^. NE is mediated to accumulate around the tumors by CXC inflammatory chemokines secreted by cancer cells^28^, ^29^. At first, NE was considered as an anti-tumor defense factor of the immune system, but according to more and more experimental evidence, NE could be polarized into phenotypes with different functions. Tumor associated neutrophils (TANs) promote tumor growth, invasion and metastasis by releasing multiple immune regulatory and angiogenic factors including vascular growth factor (VEGF), interleukin-1 (IL-1) and tumor necrosis factor (TNF)^30^-^32^. Recently there were some research suggested that neutrophil extracellular traps (NETs) involved in promoting cancer cell migration through the trap circulating tumoral cells^33^. Similarly, MONO could be differentiated into tumor associated macrophages (TAMs) in the tumor microenvironment, and recruit to tumors through many biological molecules^34^-^36^. Duo to the TAMs, expression of growth factors, matrix proteases, promotion of angiogenesis and suppression of adaptive immunity were adopted to promote tumor function^37^. Based on the current understanding of the pro-cancer mechanism about tumor inflammatory microenvironment, relevant research projected that anti-inflammatory drugs represented by NSAID could effectively inhibit the progress of colorectal cancer were being widely carried out and had obtained strong support from cell and molecular experiments^38^-^40^. On the other hand, antitumor immune activity depends mainly on the defense barrier of lymphocytes. CD4+T and CD8+T cells assume tumor immunity through induction of perforin and Granzyme B secretion, activation of NK cells and direct killing. Low levels of lymphocytes often indicate tumor immunosuppression^41^, ^42^. NLR and LMR are the most widely studied indicators of inflammatory response in the body, which comprehensively reflects the changes of inflammatory state when the body's anti-tumor immune balance is broken and eventually evolves into tumor progression. Compared with the evaluation of individual indicators, NLR and LMR can evaluate the imbalance of the tumor-immune system in CRCs more comprehensively. EO, a kind of inflammatory mature white blood cells, was concerned in allergic, blood and autoimmune diseases^43^, ^44^. In the previous concept, the content of EO in peripheral blood was small and unstable, so its value in study of cancers was ignored. As an important part of the immune response, following activation of EO release a variety of inflammatory factors that promote the progression of inflammation and cause tissue damage^45^. We found that EO was also a very good independent prognostic factor, so when designing IRR, we added EO into the formula and obtained a more accurate IRR index rationally. Then we concluded that in future CRC-related inflammation studies, more attention should be paid to this group of classic inflammatory factors with low content in peripheral blood but not negligible.

Preoperative anemia is common in various cancers. Patients with CRC, commonly due to iron-deficiency, have more significant preoperative anemia, which is a high-risk factor for poor prognosis^46^. CRC tumors can directly affect the absorption function of digestive tract. Moreover, intestinal obstruction and/or chronic colorectal bleeding caused by cancer progression are often closely related to the serious decline of patients' physical state. At the same time, chronic inflammatory is also a pathogenesis for normocytic anemia and microcytic anemia in CRC^47^, ^48^. The current medical challenge is that immunosuppression caused by red blood cell transfusion could induce tumor recurrence^49^, ^50^. In present study, HGB and MCHC in peripheral blood of CRCs were low, indicating significant anemia, accompanied by abnormally high RDW-CV. The closely related MCV, MCH and MCHC reflect the anemia degree from the volume and deformation of red cell 51. MCHC is calculated according to MCH and MCV results (MCHC=MCH/MCV), and showed the optimal sensitivity and specificity with CRC prognostic risk, so we selected MCHC to be included in our ARR formula. Kato et al reported that^52^, MCV was an independent predictor irrespective of the location of the tumor, whereas anemia symptom was not an independent predictor. Other studies suggest otherwise^46^, lower MCV was associated with advanced T stage and proximal tumor location, while HGB in patients with tumors in proximal colon was significantly lower relative to distal colon and rectum. However, Wilson's meta-analysis pointed out that long-term reduction of OS and DFS was significantly correlated with preoperative anemia in rectal cancers, but not in colon cancers^53^. Our study supported the view that indicators including HGB, MCV, and MCHC were worse in colon cancers. Previous reports, including ours, had highlighted the adverse prognostic warning of CRC at excessive RDW-CV level^17^, ^54^. Based on the previous conclusions, the results of this study again utilized and expanded the significance of high RDW-CV in peripheral blood of CRC.

CRC studies with PLT, PDW, MPV and other platelet-related indicators as the study objects had emerged one after another in recent years^10^, ^55^. Platelets not only produce the blood clotting cascade, but also regulate inflammatory response and cancer pathogenesis. Activating platelets can promote tumor growth, angiogenesis and invasion^56^. Published findings supported that PLT changes in CRC were based on systemic inflammation, but were inconclusive as a risk factor for prognosis and survival^57^, ^58^. PLR is a relatively concerned PLT-related index, which was believed to be associated with the prognosis of CRC by some scholars^59^, ^60^. The significance of PDW prognostic risk factors varied among different cancers: In breast cancer elevated PDW was considered a marker of poor prognosis^61^; Reduced PDW was an unfavorable predictive factor in non-small cell lung cancers and gastric cancers^62^, ^63^. The role of PDW in CRC was rarely studied. Our study found that PDW, as a protective factor, was inversely correlated with the progression of CRC. That was, increased PDW indicated a good prognosis, while decreased PDW indicated poor DFS and OS. We developed CRR indexes using PLT and PDW, which could effectively indicate the prognosis risk of CRC. But the risk assessment significance of CRR was the worst of the six indexes discussed in our study. We recommend it as a supplementary evaluation index. Nevertheless, we emphasize again that the change in PDW in CRC progression deserves noting and further study.

At the same time, in our study, the characteristics of invasive growth, serous membrane and adhesion of CRC were associated with abnormalities of many indicators. The severe local inflammatory changes caused by these high-risk factors could explain the reasons for the different levels of peripheral blood indicators and also support the currently recognized pro-cancer mechanism of inflammatory-cancers.

In recent years, studies on comprehensive indicators of CRC patients' peripheral blood had attracted more and more attention. Relevant research contents were more detailed, or applied to clinical antitumor therapy^22^, ^64^. Chen et al. discussed the relationship between NLR and LMR in patients with obstructive colorectal cancer (OCC) who received emergency surgery or self-expandable metal stents (SEMS), and concluded that OCC patients with low LMR might be preferred for SEMS insertion as a bridge to surgery^65^. In terms of chemotherapy, studies had suggested that low NLR patients treated with bevacizumab and low PLR patients treated with anti-EGFR had better prognosis and NLR was a prognostic biomarker for CRC patients receiving TAS- 102 treatment^66^, ^67^. In our study, three new comprehensive indexes of peripheral blood (IRR, ARR, CRR) were obtained by using novel calculation design. They had good prognostic evaluation efficiency and could also reflect the changes of general states of CRCs from different aspects. We hoped that these indexes, especially IRR, can be applied to more in-depth and detailed CRC studies for validation in the future work.

## Conclusion

In this study, appropriate indicators were selected from the BRT results of CRC patients to design IRR, ARR and CRR, three CRC prognostic risk indexes related to infection, anemia and coagulation in peripheral blood. All of them had satisfactory prognostic risk warning ability for DFS and OS, among which IRR was the most effective and better than NLR, PLR and LMR. Abnormalities in these indexes might be due to local inflammatory responses, immunosuppression, and associated anemia and platelet problems. It could be concluded that these three indexes, represented by IRR, could be used as CRC prognostic risk indicators with the advantages of simple, effective, low-cost and repeatable.

## Supplementary Material

Supplementary tables.

## Figures and Tables

**Table 1:** Clinicopathological characteristics of patients with CRC

Characteristic	Number of cases	%
**Total**	701	100
**Average age, years (range)**	61.8 (24-91)	
**Age (years)**		
≤61	342	48.8
>61	359	51.2
**Gender**		
Male	428	61.1
Female	273	38.9
**Tumor location**		
Colon (Right/Left)	227 (89/138)	32.4
Rectum	474	67.6
**TNM stage**		
I	111	15.8
II	292	41.7
III	262	37.4
IV	36	5.1
**Infiltration**		
Invaded serosa (T1, T2, T3)	251	35.8
Non‑invaded serosa (T4)	450	64.2
**Lymphatic node**		
Negative (N0)	422	60.2
Positive (N1, N2)	279	39.8
**Metastasis**		
Negative (M0)	665	94.9
Positive (M1)	36	5.1
**Differentiation**		
Poorly	130	18.5
Medium	384	54.8
Well	187	26.7
**Pathological pattern**		
Adenocarcinoma	552	78.7
Non-adenocarcinoma	149	21.3
**Morphology**		
Protuberant type	147	21.0
Ulcerative/Invasive type	554	79.0
**Growth pattern**		
Protuberant/Nest growth	290	41.4
Ulcerative/Invasive growth	411	58.6
**Adhesion**		
Negative	598	85.3
Positive	103	14.7
**Disease free survival (DFS)**		
Non-advanced	517	73.8
Advanced	184	26.2
**Overall survival (OS)**		
Survival	523	74.6
Dead	178	25.4
**Follow-up, days (range)**		
DFS	989.3 (10-1973)	
OS	1199.8 (10-1973)	

**Table 2:** Correlation of BRT results to CRC prognosis

Characteristics		Inflammatory related factors						Anemia related factors								Cruor related factors				
		WBC	LY	NE	MONO	EO	BASO	RBC	HGB	HCT	MCV	MCH	MCHC	CV	SD	PLT	PDW	P-LCR	PCT	MPV
DFS	r	0.041	-.151^**^	.101^**^	.108^**^	-.126^**^	-0.045	-.154^**^	-.178^**^	-.174^**^	-.080^*^	-.110^**^	-.113^**^	.143^**^	.099^**^	.074^*^	-.096^*^	-.076^*^	0.051	-0.072
	*P*	0.282	**0.000**	**0.008**	**0.004**	**0.001**	0.231	**0.000**	**0.000**	**0.000**	**0.034**	**0.004**	**0.003**	**0.000**	**0.009**	**0.049**	**0.011**	**0.046**	0.175	0.058
OS	r	0.030	-.129^**^	.076^*^	.093^*^	-.117^**^	-0.050	-.136^**^	-.171^**^	-.162^**^	-.081^*^	-.109^**^	-.126^**^	.189^**^	.143^**^	.077^*^	-.089^*^	-0.068	0.073	-0.064
	*P*	0.431	**0.001**	**0.044**	**0.014**	**0.002**	0.188	**0.000**	**0.000**	**0.000**	**0.033**	**0.004**	**0.001**	**0.000**	**0.000**	**0.041**	**0.019**	0.071	0.054	0.091

* P<0.05, **P <0.01

**Table 3:** Interactions between IRR/ARR/CRR/NLR/PLR/LMR and CRC characteristics

Indexes	TNM			Infiltration			Adhesion			DFS			OS		
	HR	95%CI	*P*	HR	95%CI	*P*	HR	95%CI	*P*	HR	95%CI	*P*	HR	95%CI	*P*
IRR	1.726	1.112-2.678	**0.015**	1.409	1.023-1.941	**0.036**	2.151	1.409-3.284	**0.000**	2.738	1.929-3.885	**0.000**	2.506	1.760-3.569	**0.000**
ARR	2.096	1.272-3.453	**0.004**	2.165	1.502-3.120	**0.000**	3.176	2.025-4.982	**0.000**	2.496	1.724-3.614	**0.000**	2.726	1.869-3.974	**0.000**
CRR	3.226	1.526-6.819	**0.002**	2.348	1.483-3.717	**0.000**	2.455	1.530-3.939	**0.000**	2.235	1.477-3.381	**0.000**	2.520	1.660-3.825	**0.000**
NLR	1.788	1.158-2.760	**0.009**	1.855	1.344-2.560	**0.000**	2.555	1.655-3.946	**0.000**	2.083	1.473-2.946	**0.000**	1.946	1.371-2.763	**0.000**
PLR	1.552	1.030-2.341	**0.036**	1.684	1.231-2.304	**0.001**	4.222	2.543-7.011	**0.000**	2.278	1.591-3.262	**0.000**	2.138	1.488-3.071	**0.000**
LMR	2.512	1.510-4.176	**0.000**	1.697	1.206-2.387	**0.002**	2.914	1.888-4.498	**0.000**	2.270	1.594-3.231	**0.000**	2.053	1.437-2.933	**0.000**

*P* values were calculated by unconditional logistic regression adjusted for age and gender.

**Table 4:** Multivariate Cox proportional hazard analyses of IRR, ARR, CRR, NLR, PLR and LMR in train set

Index	DFS				OS		
	Adjusted HR	95% CI	*P* value*		Adjusted HR	95% CI	*P* value
IRR	2.106	1.566-2.832	**0.000**		1.931	1.433-2.603	**0.000**
ARR	1.747	1.279-2.386	**0.000**		1.884	1.370-2.590	**0.000**
CRR	1.679	1.211-2.330	**0.002**		1.798	1.295-2.496	**0.000**
NLR	1.651	1.228-2.219	**0.001**		1.538	1.139-2.078	**0.005**
PLR	1.935	1.409-2.657	**0.000**		1.777	1.291-2.446	**0.000**
LMR	1.860	1.380-2.507	**0.000**		1.637	1.208-2.217	**0.001**

*P values were calculated by multivariate cox proportional hazard analyses adjusted for age, gender, TNM stage, infiltration and adhesion.

## Abbreviations

CRC: Colorectal Cancer
BRT: Blood Routine Test
NLR: Neutrophil-to-Lymphocyte Ratio
PLR: Platelet-to-Lymphocyte Ratio
LMR: Lymphocyte-to-Monocyte ratio
WBC: Signeta-Ring Cell Carcinoma
WBC: White Blood Cell
LY: Lymphocyte
NE: Neutrophil
MONO: Monocyte
EO: Eosinophilic Granulocyte
BASO: Basophilic Granulocyte
RBC: Red Blood Cell
HGB: Hemoglobin
HCT: Hematocrit
MCV: Mean Corpuscular Volume
MCH: Mean Corpuscular Hemoglobin
MCHC: Mean Corpuscular Hemoglobin Concentration
RDW-CV: Red Cell Distribution Width-CV
RDW-SD: Red Cell Distribution Width-SD
PLT: Platelets
PDW: Platelet Distribution Width
P-LCR: Platelet-Larger Cell Ratio
PCT: Platelet Cell Thrombocytocrit
MPV: Mean Platelet Volume
IRR: Inflammatory Related Ratio
ARR: Anemia Related Ratio
CRR: Coagulation Related Ratio
TANs: Tumor Associated Neutrophils
TAMs: tumor associated macrophages
