# Supplementary material for: Prognostic evaluation of colorectal cancer using three new comprehensive indexes related to infection, anemia and coagulation derived from peripheral blood
Source: J Cancer. 2020 Apr 6;11(13):3834–45. doi: 10.7150/jca.42409 (PMC7171501; doi:10.7150/jca.42409)
Supplement: Supplementary file 1 — Supplementary tables. [file jcav11p3834s1.pdf]

Table.S1 BRT results of CRCs

Characteristic	Average value	Range
White Blood Cell (WBC, 10^9/L)	6.70	2.02-18.92
Lymphocyte (LY, 10^9/L)	1.87	0.22-5.71
Neutrophil (NE, 10^9/L)	4.18	0.94-17.96
Monocyte (MONO, 10^9/L)	0.45	0.14-1.77
Eosinophilic Granulocyte (EO, 10^9/L)	0.16	0.01-1.56
Basophilic Granulocyte (BASO, 10^9/L)	0.03	0.00-0.13
Red Blood Cell (RBC, 10^{12}/L)	4.61	2.26-6.37
Hemoglobin (HGB, g/L)	134.52	35.00-185.00
Hematocrit (HCT, L/L)	0.41	0.14-0.55
Mean Corpuscular Volume (MCV, fL)	88.01	51.00-108.50
Mean Corpuscular Hemoglobin (MCH, pg)	29.14	15.00-37.00
Mean Corpuscular Hemoglobin Concentration (MCHC, gL)	330.24	250.00-381.00
Red Cell Distribution Width-CV (RDW-CV, %)	13.39	11.00-32.60
Red Cell Distribution Width-SD (RDW-SD, fL)	42.72	35.00-77.00
Platelets (PLT, 10^9/L)	246.39	73.00-590.00
Platelet Distribution Width (PDW, 10GSD)	11.86	7.90-21.80
Platelet-Larger Cell Ratio (P-LCR, %)	27.18	8.20-55.90
Platelet Cell Thrombocytocrit (PCT, L/L)	0.26	0.10-0.50
Mean Platelet Volume (MPV, fL)	10.31	7.80-14.00

Table.S2 Correlation of BRT results to CRC characteristics

Characteristics		Inflammatory related factors						Anemia related factors								Cruor related factors				
		WBC	LY	NE	MONO	EO	BASO	RBC	HGB	HCT	MCV	MCH	MCHC	CV	SD	PLT	PDW	P-LCR	PCT	MPV
Age	r	-0.006	**-.081***	0.007	0.055	-0.008	**-.103****	**-.226****	**-.217****	**-.199****	0.013	**-.078***	**-.171****	**.194****	**.229****	-0.039	-0.008	-0.009	-0.063	-0.008
	P	0.865	**0.032**	0.844	0.144	0.833	**0.006**	**0.000**	**0.000**	**0.000**	0.731	**0.039**	**0.000**	**0.000**	**0.000**	0.298	0.824	0.814	0.098	0.842
Gender	r	**.084***	-0.003	**.077***	**.177****	**.132****	0.047	**.348****	**.436****	**.406****	**.196****	**.303****	**.296****	**-.093***	0.034	**-.152****	-0.062	**-.092***	**-.166****	**-.087***
	P	**0.027**	0.938	**0.041**	**0.000**	**0.000**	0.210	**0.000**	**0.000**	**0.000**	**0.000**	**0.000**	**0.000**	**0.013**	0.365	**0.000**	0.102	**0.015**	**0.000**	**0.021**
Tumor location	r	-0.017	**.079***	-0.032	-0.044	0.046	0.008	**.179****	**.273****	**.269****	**.242****	**.231****	**.198****	**-.195****	-0.019	**-.138****	0.027	0.027	**-.133****	0.037
	P	0.650	**0.037**	0.393	0.240	0.226	0.837	**0.000**	**0.000**	**0.000**	**0.000**	**0.000**	**0.000**	**0.000**	0.613	**0.000**	0.474	0.475	**0.000**	0.326
Colon location	r	-0.089	**-0.148**	-0.036	-0.130	-0.049	-0.001	0.002	0.015	0.039	0.038	0.005	-0.042	0.087	0.099	-0.055	0.035	0.019	-0.015	0.007
	P	0.184	**0.026**	0.588	0.051	0.459	0.987	0.974	0.819	0.558	0.572	0.945	0.524	0.190	0.138	0.413	0.598	0.776	0.818	0.919
TNM stage	r	0.067	**-.080***	**.111****	**.095***	-0.038	0.051	-0.041	**-.074***	-0.074	**-.102****	**-.109****	**-.093***	**.108****	0.029	**.092***	**-.074***	-0.064	0.064	-0.069
	P	0.077	**0.035**	**0.003**	**0.012**	0.310	0.175	0.276	**0.049**	0.051	**0.007**	**0.004**	**0.013**	**0.004**	0.448	**0.015**	**0.049**	0.090	0.090	0.068
Infiltration	r	0.057	**-.082***	**.101****	**.085***	0.022	**.099****	-0.044	**-.119****	**-.103****	**-.135****	**-.155****	**-.141****	0.060	-0.012	**.130****	-0.032	-0.031	**.119****	-0.037
	P	0.133	**0.030**	**0.007**	**0.024**	0.565	**0.009**	0.242	**0.002**	**0.006**	**0.000**	**0.000**	**0.000**	0.113	0.750	**0.001**	0.397	0.419	**0.002**	0.322
Lymphatic node	r	0.019	**-.084***	0.049	0.042	-0.050	0.054	-0.027	-0.034	-0.038	-0.055	-0.067	-0.056	0.062	0.027	0.028	-0.055	-0.043	0.002	-0.048
	P	0.614	**0.026**	0.197	0.263	0.188	0.154	0.476	0.363	0.315	0.146	0.075	0.142	0.098	0.469	0.463	0.143	0.258	0.949	0.204
Metastasis	r	**.074***	-0.021	**.097****	**.090***	-0.008	0.013	**-.081***	**-.076***	-0.072	-0.011	-0.033	-0.053	0.065	0.052	0.068	**-.080***	-0.058	0.052	-0.056
	P	**0.049**	0.576	**0.010**	**0.017**	0.830	0.741	**0.031**	**0.044**	0.057	0.769	0.378	0.161	0.087	0.171	0.073	**0.035**	0.125	0.168	0.136
Pathological	r	0.034	-0.040	0.064	0.027	-0.028	-0.010	-0.041	**-.102****	**-.089***	**-.143****	**-.152****	**-.102****	0.034	-0.023	0.053	-0.025	-0.022	0.033	-0.026
pattern	*P*	0.365	0.286	0.092	0.474	0.452	0.801	0.277	**0.007**	**0.019**	**0.000**	**0.000**	**0.007**	0.374	0.539	0.163	0.517	0.558	0.384	0.497

Differentiation	r	0.009	0.011	0.012	-0.047	0.014	0.038	-0.008	0.008	-0.003	-0.033	-0.002	0.026	-0.004	-0.028	-0.021	-0.007	-0.002	-0.008	-0.004
	P	0.803	0.764	0.751	0.218	0.717	0.309	0.826	0.837	0.947	0.390	0.951	0.493	0.907	0.461	0.581	0.861	0.962	0.824	0.918
Morphology	r	0.019	-0.034	0.045	0.066	0.008	0.001	-0.009	-0.035	-0.028	-0.024	-0.028	-0.066	0.070	0.052	**.097***	**-.116****	**-.116****	0.052	**-.116****
	P	0.610	0.363	0.236	0.079	0.840	0.972	0.802	0.358	0.465	0.529	0.453	0.082	0.064	0.169	**0.010**	**0.002**	**0.002**	0.170	**0.002**
Growth pattern	r	0.015	-0.029	0.050	-0.025	**-.077***	0.023	0.053	0.006	0.022	-0.056	-0.065	-0.038	-0.030	-0.060	0.046	0.006	-0.014	0.048	-0.016
	P	0.696	0.442	0.190	0.504	**0.041**	0.540	0.161	0.878	0.559	0.137	0.085	0.310	0.423	0.112	0.225	0.882	0.703	0.201	0.679
Adhesion	r	**.091***	**-.133****	**.139****	**.128****	-0.051	0.006	**-.103****	**-.166****	**-.169****	**-.195****	**-.199****	**-.144****	**.177****	0.020	**.153****	**-.098****	**-.103****	**.112****	**-.109****
	P	**0.016**	**0.000**	**0.000**	**0.001**	0.175	0.878	**0.006**	**0.000**	**0.000**	**0.000**	**0.000**	**0.000**	**0.000**	0.601	**0.000**	**0.009**	**0.006**	**0.003**	**0.004**

* $P<0.05$

**$P<0.01$

Table.S3 Kaplan-Meier survival analysis results of six indexes in train set

Indexes	DFS		OS	
	HR (95%CI)	*P* value*	HR (95%CI)	*P* value
IRR				
Normal	1639.2 (1580.0-1698.3)	**<0.001***	1696.6 (1644.2-1749.1)	**<0.001***
Abnormal	1357.2 (1267.0-1447.4)		1459.8 (1379.4-1540.2)	
ARR				
Normal	1645.3 (1586.0-1704.7)	**<0.001***	1701.1 (1649.7-1752.4)	**<0.001***
Abnormal	1319.7 (1225.9-1413.5)		1415.9 (1330.3-1501.5)	
CRR				
Normal	1591.1 (1534.8-1647.5)	**<0.001***	1657.0 (1608.4-1705.6)	**<0.001***
Abnormal	1281.6 (1148.3-1414.9)		1357.2 (1234.9-1479.4)	
NLR				
Normal	1653.0 (1589.4-1716.5)	**<0.001***	1698.7 (1643.8-1753.7)	**<0.001***
Abnormal	1352.0 (1271.2-1432.8)		1442.2 (1370.0-1514.4)	
PLR				
Normal	1679.8 (1613.1-1746.6)	**<0.001***	1719.9 (1662.1-1777.8)	**<0.001***
Abnormal	1380.7 (1305.4-1456.0)		1472.3 (1406.3-1538.3)	
LMR				
Normal	1648.0 (1588.4-1707.6)	**<0.001***	1695.1 (1643.9-1746.3)	**<0.001***
Abnormal	1310.4 (1216.0-1404.8)		1415.2 (1329.7-1500.7)	

***P* values were calculated by multivariate cox proportional hazard analyses adjusted for age, gender, TNM stage, infiltration and adhesion.

Table.S4 Kaplan-Meier survival analysis results of three indexes in test set

Indexes	DFS		OS	
	HR (95%CI)	*P* value*	HR (95%CI)	*P* value*
IRR				
Normal	1698.5 (1614.3-1782.7)	**<0.001***	1729.9 (1650.0-1809.8)	**<0.001***
Abnormal	1217.0 (1064.7-1369.3)		1345.5 (1202.4-1488.7)	
ARR				
Normal	1670.9 (1576.0-1765.9)	**<0.001***	1713.2 (1628.0-1798.5)	**<0.001***
Abnormal	1256.8 (1103.0-1410.6)		1340.1 (1204.3-1475.9)	
CRR				
Normal	1602.9 (1510.7-1695.1)	**<0.001***	1652.6 (1572.5-1732.7)	**<0.001***
Abnormal	1154.6 (924.5-1384.7)		1227.9 (1007.7-1448.0)	

***P* values were calculated by multivariate cox proportional hazard analyses adjusted for age, gender, TNM stage, infiltration and adhesion.

Table.S5 Multivariate Cox proportional hazard analyses of IRR, ARR and CRR in test set

Index	DFS			OS		
	Adjusted HR	95% CI	*P* value*	Adjusted HR	95% CI	*P* value
IRR	3.674	2.120-6.367	**0.000**	3.123	1.854-5.259	**0.000**
ARR	2.131	1.255-3.618	**0.005**	2.268	1.315-3.910	**0.003**
CRR	2.467	1.452-4.192	**0.001**	2.370	1.400-4.013	**0.001**

*****_P_ values were calculated by multivariate cox proportional hazard analyses adjusted for age, gender, TNM stage, infiltration and adhesion.
